# P10 Phage-encoded lysins as promising antibacterials against uropathogenic *Escherichia coli*

**DOI:** 10.1093/jacamr/dlac133.014

**Published:** 2023-01-19

**Authors:** Sarmad Hanif, Ritam Das, Bhakti Chavan, Sanket Shah, Urmi Bajpai, Syed Ahmed

**Affiliations:** Techinvention Lifecare Pvt. Ltd, Mumbai, India; Techinvention Lifecare Pvt. Ltd, Mumbai, India; Techinvention Lifecare Pvt. Ltd, Mumbai, India; Techinvention Lifecare Pvt. Ltd, Mumbai, India; Techinvention Lifecare Pvt. Ltd, Mumbai, India; Techinvention Lifecare Pvt. Ltd, Mumbai, India; Acharya Narendra Dev College, University of Delhi, New Delhi, India

## Abstract

**Background:**

Urinary tract infections (UTIs) represent a major public health issue. Uropathogenic *Escherichia coli* (UPEC) is a pathogen implicated in the vast majority of UTIs. Although treatable, the rampant and indiscriminate use of antibiotics has led to an alarming emergence of MDR strains. This can lead to complications, treatment failure, and increased rates of mortality and morbidity. Given the prevalence of these strains, we are exploring phage encoded-lysins as potential alternatives to antibiotics.

**Objectives:**

Our study involved the development of an *in silico* strategy for the discovery and characterization of prophage-derived lysin sequences targeting the *E. coli* cell wall and evaluating the antibacterial activity of the recombinant lysins using *in vitro* assays.

**Methods:**

Novel lysin sequences were searched by BLAST homology and by screening *E. coli* prophages in the database (using PHASTER). They were computationally characterized and their domain architecture was examined. Based on the desired physicochemical properties of the 16 lysins, 7 were selected for cloning, expression, and purification as recombinant proteins for the *in vitro* antibacterial activity.

**Results:**

*In silico* analysis showed lysozyme-like domain in 9 out of 16 lysins and the predicted structure was either modular or globular. Cationic and hydrophobic residues at the C-terminal end and cationic residues in the catalytic domains indicate intrinsic bactericidal activity. Lysins (7) were cloned in pET28 vector and purified as His-tagged recombinant proteins by Ni-NTA chromatography. Using spot assay, 3 out of 7 lysins (9–12 μg) showed lysis of *E. coli* cells (BL21 DE3). The most promising was lysin sequence 5, which showed lysis of *E. coli* cells, both at stationary as well as mid-log phase cells. Turbidity reduction assay showed a 74.94% drop in OD600 in lysin (15 μM) treated cells after 3 h of incubation, at 37°C. Log killing assay showed 4 log_10_ reduction in the treated cells. Lysozyme assay demonstrates higher than / comparable lysozyme activity of lysin sequence 5 with the positive control.

**Conclusions:**

Sixteen novel prophage-encoded lysin sequences have been identified to create a lysin bank (to screen against *E. coli*), and 7 of them have been purified. Screening shows three lysins having intrinsic bactericidal activity and hence did not require the addition of an Outer Membrane Permeabilizer (OMP). Lysin sequence 5 has exhibited the highest activity against *E. coli* cells. Enzyme stability and kinetics studies and screening of clinical isolates of *E. coli* from UTI patients is underway. Investigating this bank of lysins is aimed at the identification and development of a lead ‘enzybiotic’ with desired PK/PD characteristics against UTI-causing *E. coli*.

